# Epilepsy and neuropsychiatric comorbidities in mice carrying a recurrent Dravet syndrome *SCN1A* missense mutation

**DOI:** 10.1038/s41598-019-50627-w

**Published:** 2019-10-02

**Authors:** Ana Ricobaraza, Lucia Mora-Jimenez, Elena Puerta, Rocio Sanchez-Carpintero, Ana Mingorance, Julio Artieda, Maria Jesus Nicolas, Guillermo Besne, Maria Bunuales, Manuela Gonzalez-Aparicio, Noemi Sola-Sevilla, Miguel Valencia, Ruben Hernandez-Alcoceba

**Affiliations:** 1 University of Navarra, Gene Therapy Program CIMA, IdiSNA, Navarra institute for health research, Pamplona, Spain; 2 University of Navarra, Department of Pharmacology and Toxicology, IdiSNA, Navarra institute for health research, Pamplona, Spain; 3 University Clinic of Navarra, Dravet Syndrome Unit, Pediatric Neurology Unit, IdiSNA, Navarra institute for health research, Pamplona, Spain; 4 Dracaena Consulting, Madrid, Spain; 5 University of Navarra, Neuroscience Program CIMA, IdiSNA, Navarra institute for health research, Neurophysiology Service, Clinica Universidad de Navarra, University of Navarra, Pamplona, Spain; 6 University of Navarra, Neuroscience Program CIMA, IdiSNA, Navarra institute for health research, Pamplona, Spain

**Keywords:** Epilepsy, Epilepsy

## Abstract

Dravet Syndrome (DS) is an encephalopathy with epilepsy associated with multiple neuropsychiatric comorbidities. In up to 90% of cases, it is caused by functional happloinsufficiency of the *SCN1A* gene, which encodes the alpha subunit of a voltage-dependent sodium channel (Nav1.1). Preclinical development of new targeted therapies requires accessible animal models which recapitulate the disease at the genetic and clinical levels. Here we describe that a C57BL/6 J knock-in mouse strain carrying a heterozygous, clinically relevant *SCN1A* mutation (A1783V) presents a full spectrum of DS manifestations. This includes 70% mortality rate during the first 8 weeks of age, reduced threshold for heat-induced seizures (4.7 °C lower compared with control littermates), cognitive impairment, motor disturbances, anxiety, hyperactive behavior and defects in the interaction with the environment. In contrast, sociability was relatively preserved. Electrophysiological studies showed spontaneous interictal epileptiform discharges, which increased in a temperature-dependent manner. Seizures were multifocal, with different origins within and across individuals. They showed intra/inter-hemispheric propagation and often resulted in generalized tonic-clonic seizures. ^18^F-labelled flourodeoxyglucose positron emission tomography (FDG-PET) revealed a global increase in glucose uptake in the brain of Scn1a^WT/A1783V^ mice. We conclude that the Scn1a^WT/A1783V^ model is a robust research platform for the evaluation of new therapies against DS.

## Introduction

Dravet syndrome (DS) is a severe early onset encephalopathy (OMIM 607208) with an average incidence of 1:20,000 births. Although it is also known as severe myoclonic epilepsy of infancy (SMEI), accounting for 1.4% of children with epilepsy^1^, seizures are not the only manifestations of the disease^2^. The first symptoms start at 4–8 months of age, in a previously normal infant, as clonic or hemiclonic febrile seizures, usually prolonged and refractory to conventional antiepileptic drugs. The frequency of life-threatening status epilepticus (SE) in these patients can reach 80% during the first year of life. After this “febrile stage” of the disease, a “worsening stage” extends up to the fifth year of life, in which afebrile myoclonic, focal or generalized seizures are frequent, as well as atypical absences^3^. During this period a variety of neurological disturbances appear, including psychomotor delay leading to cognitive disability, motor disturbances as well as hyperkinesia and some features of autism spectrum disorder. During the “stabilization stage” these invalidating problems persist and crouching gait as well as parkinsonian features appear, but epileptic episodes are less frequent. However, the risk of sudden unexpected death in epilepsy (SUDEP) is always present. Overall, mortality rate in DS is estimated at 15% in industrialized countries^4^, with approximately 50% of cases due to SUDEP and 35% due to SE.

In nearly 90% of DS patients, the genetic basis of the disease involves the *SCN1A* gene^5,6^, which encodes the alpha subunit of a voltage-dependent sodium channel (Nav1.1). This membrane transporter is crucial for the function of GABAergic inhibitory interneurons expressing parvalbumin or somatostatin (PV and ST cells, respectively)^7,8^. Insufficient Nav1.1 activity causes alteration in the excitatory/inhibitory balance of the brain, which is the basis for most clinical manifestations.

Homozygous deletions or loss of function mutations of *SCN1A* are extremely rare in humans, probably because of embryonic lethality. DS patients usually present heterozygous mutations resulting in functional inactivation of one *SCN1A* allele^9^. In approximately half of the cases, the mutated allele produces a truncated Nav1.1 channel due to nonsense, frameshift or splice defect mutations (17%, 19% and 9%, respectively)^10^. The other half presents missense mutations or in-frame deletions, with variable impact on channel function. In the lower spectrum of severity, missense mutations causing moderate impairment have been associated with milder diseases such as genetic epilepsy with febrile seizures plus (GEFS^+^)^11^. In addition, diverse functional alterations of Nav1.1 may contribute to other neurological disorders such as autism^12^, familial hemiplegic migraine^13^, and aging-related cerebral impairment^14^.

Advances in the understanding of DS pathophysiology and the development of new therapies require relevant animal models. This is especially important for the evaluation of novel approaches aimed at restoring Nav1.1 expression or function, which offer the opportunity to control not only seizures, but also the rest of invalidating comorbidities.

A variety of genetic mouse models based on *Scn1a* alterations have been described. Most of them rely on deletions of the gene, either globally or affecting specific cell populations. Mice harboring homozygous deletions in the C57BL/6 background inexorably die two weeks after birth^15–17^, whereas happloinsufficient mice show spontaneous seizures and elevated mortality from 3 to 12 weeks of age, resulting in 20% long-term survival^16^. In contrast, clinical manifestations are very mild in the 129 Sv background^18^. Conditional deletion of *Scn1a* in different cell populations has been obtained by crossing mice carrying one floxed *Scn1a* allele with those expressing Cre recombinase under the control of specific promoters^7,19–21^. The prominent role of GABAergic inhibitory interneurons is in line with the drastic epileptic phenotype observed in the VGAT-Cre strain, which is more severely affected than the global *Scn1a* deficient mice. In contrast, deletion of *Scn1a* in excitatory neurons (Emx1-Cre strain) showed no epileptic phenotype^19^. Supporting this concept, a recent work demonstrates that 100% of mice carrying the *Scn1a* A1783V mutation in VGAT-expressing cells die before postnatal day 25^22^. To narrow down the implication of different interneuron populations, Nav1.1 was deleted in PV *vs* ST cells. The results showed that defects in ST cells caused only a mild phenotype. In contrast, mice with defects in PV cells suffered a marked reduction in the threshold temperature for hyperthermia-induced seizures, together with behavioral abnormalities^20^. However, the implication of ST cells in the phenotype of DS mice has been demonstrated in other reports^7^. Apart from the specific alteration introduced in the *Scn1a* gene of each mouse model (deletions, frameshift or missense mutations), the genetic background is an important modifier factor^16,18^. This may explain discrepancies between different studies regarding the role of ST and PV cells on specific manifestations such as hyperactivity and autistic-like behaviors^7,20^. Although these tools are helping to elucidate the physiopathology of DS, preclinical development of new therapies requires a widely available mouse model with the ability to recapitulate the human disease at the genetic and phenotypic levels.

To this end, we have employed conditional knock-in mice with a heterozygous *Scn1a* A1783V mutation in all cells, maintained in a C57BL/6J background. This is a pathogenic missense mutation in exon 26, previously described in DS patients^10,23^, which is expected to affect the inactivation gate receptor of Nav1.1 located in the S6 segment of domain 4^22,24^. In this report we describe a remarkable reproduction of DS manifestations in all aspects of the disease such as epileptic activity, motor, behavioral and cognitive alterations. Of note, a knock-in model harboring the same mutation (B6(Cg)-Scn1atm1.1Dsf/J strain crossed with Cox2-Cre expressing mice) has been recently adopted by the US National Institute of Neurological disorders and Stroke (NINDS) in the panel of animal models of the Epilepsy Therapy Screening Program (ETSP). This is the first model of genetic epilepsy to be included in the panel (https://dravet.eu/projects-item/mouse-model/).

## Results

### Scn1a^WT/A1783V^ mice have a high mortality rate

**Figure 1:**
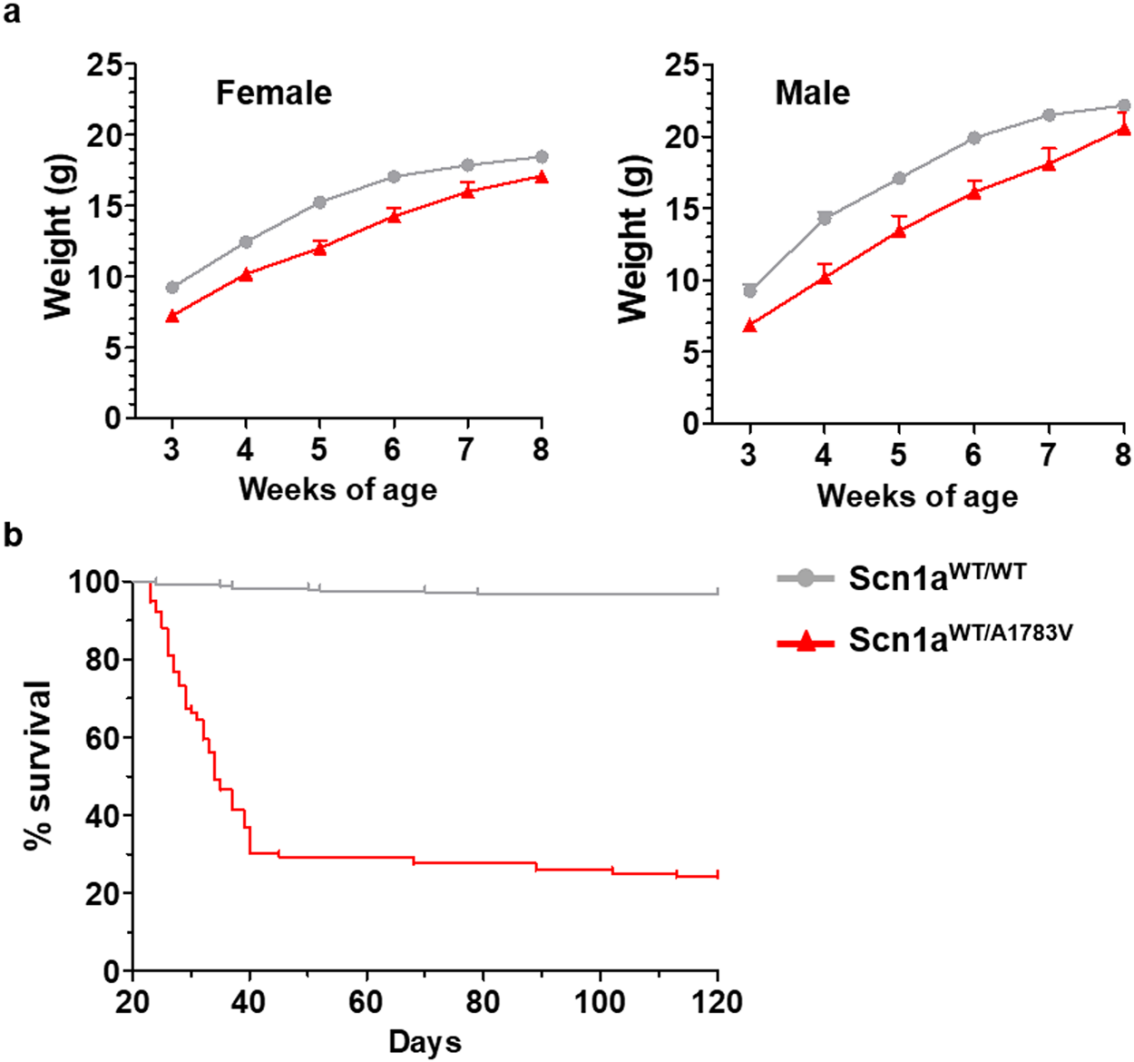
Scn1a^WT/A1783V^ mice present reduced body weight and high mortality during the first 6 weeks of life. (**a**) Animals were weighted once per week since weaning. Points represent mean values ± SEM (females Scn1a^WT/WT^ average n = 30; females Scn1a^WT/A1783V^ n = 20; males Scn1a^WT/WT^ n = 40; males Scn1a^WT/A1783V^ n = 15). Weight curves of Scn1a^WT/A1783V^ and control littermates mice show significant differences in elevation (p < 0.0001) but not in slope (linear regression analysis). (**b**) Survival curves of mice after weaning (postnatal day 21) are significantly different (p < 0.0001 log-rank test). Of note, Scn1a^WT/A1783V^ mice present a 24% mortality rate before weaning.

The B6(Cg)-*Scn1a*^*tm1.1Dsf*^/J strain contains *Scn1a* exon 26 flanked by loxP sites in one of the alleles, followed by a humanized exon 26 with a C to T change at nucleotide 5348 (https://www.jax.org/strain/026133). Mice from the B6.C-Tg(CMV-Cre)1Cgn/J strain express Cre under the control of the ubiquitous CMV promoter. When these strains are crossed, the floxed exon can suffer Cre-mediated scission and the mutated exon 26 is incorporated in the mature mRNA expressed from this allele. This happens in approximately half of the offspring (genotype hereinafter referred to as Scn1a^WT/A1783V^). In these mice, 50% of the Nav1.1 channels expressed in every cell contain the A1783V amino acid change. Litters of these crosses showed a 24% mortality rate before weaning. In all cases analyzed, dead pups corresponded to Scn1a^WT/A1783V^ mice. The weight of Scn1a^WT/A1783V^ mice surviving after weaning was lower than their Scn1a^WT/WT^ littermates until the 8^th^ week of age (Fig. 1a). Survival of Scn1a^WT/A1783V^ mice dropped sharply from weaning to the 6^th^ weeks of age, and then it remained relatively stable, resulting in approximately 25% long-term survivors (Fig. 1b). Although the main cause of death in these animals needs further study, our results point to SUDEP, since the frequency of spontaneous generalized tonic clonic seizures (15% of mice, starting from week 3 of age) cannot account for the high mortality rate observed.

**Figure 2:**
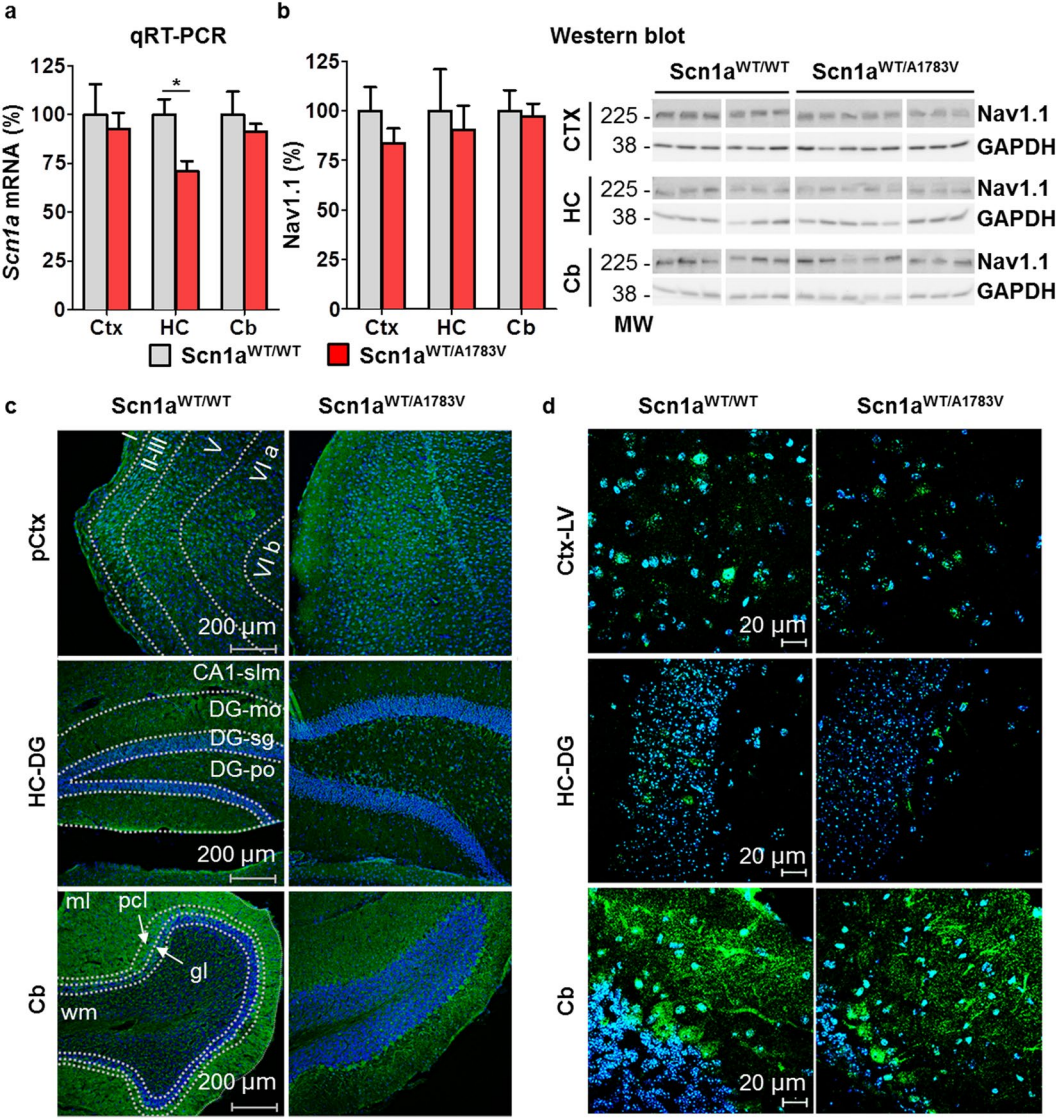
Scn1a^WT/A1783V^ mice present reduced *Scn1a* mRNA in hippocampus, but no significant differences in any brain region at the protein levels. (**a**) mRNA levels of *Scn1a* were determined by qRT-PCR in the cortex (Ctx), hippocampus (HC) and cerebellum (Cb) of Scn1a^WT/A1783V^ and aged-matched littermates (5–8 months). Values (normalized against *GAPDH* mRNA levels) were calculated as percentage *vs* control littermates and data represented as mean ± SEM (n = 6 for Scn1a^WT/WT^ mice and n = 8 for Scn1a^WT/A1783V^ mice). *p < 0.05 Kruskal-Wallis with Dunn’s post-test. (**b**) Membrane-enriched protein extracts prepared from Ctx, HC and Cb were used to evaluate Nav1.1 protein content by Western blotting. Bars represent the densitometric analysis of individual determinations normalized to GAPDH values. Data are represented as mean percentage ± SEM of values normalized to control mice (n = 6 for Scn1a^WT/WT^ mice and n = 8 for Scn1a^WT/A1783V^ mice). No statistical differences were found between control and Scn1a^WT/A1783V^ mice. The right panel corresponds to representative blots showing Nav1.1 and GAPDH bands. Original blots were cropped and re-arranged to display grouped Scn1a^WT/A1783V^ and control littermates. Full-length blots are available in supplemental material. (**c**) Additional mice were sacrificed for analysis of Nav1.1 by immunofluorescence (green). Nuclei are stained with DAPI (blue). The image shows the indicated brain areas: prefrontal cortex (pCtx); dentate gyrus of the HC (HC-DG); and Cb of representative mice (n = 4 for both groups of animals). The different regions of each structure were delimited by dotted lines based on the Allen adult mouse brain reference atlas. In pCtx roman numbers indicate its different layers. In HC-DG the following regions are included: stratum lacunosum-molecurare of CA1 (CA1-slm); and molecular layer (GD-mo), granule cell layer (DG-sg) and polymorph layer (DG-po) of dentate gyrus. In Cb: molecular layer (ml), white matter (wm), purkinje cell layer (pcl) and granule layer (gl). Scale bar 200 µm. (**d**) In order to study the subcellular localization of Nav1.1 channel, tissue sections were visualized using a confocal laser scanning microscope. The panel shows the labelling observed in cortical later V (Ctx-LV), HC-DG and Cb of representative mice (n = 4 for both groups of animals). Scale bar 20 µm.

Analysis of *Scn1a* expression quantified at the mRNA level showed a moderate reduction in hippocampus of Scn1a^WT/A1783V^ mice (Fig. 2a), but no significant changes in Nav1.1 content were observed in membrane-enriched extracts by Western blot in any brain structure. If anything, the reduction would be less than 20% (Fig. 2b), and no obvious differences were detected in the immunofluorescence analysis of brain samples (Fig. 2c,d). This is compatible with the expected outcome of a heterozygous missense mutation.

### Scn1a^WT/A1783V^ mice present abnormal interictal activity and heat-induced seizures

**Figure 3:**
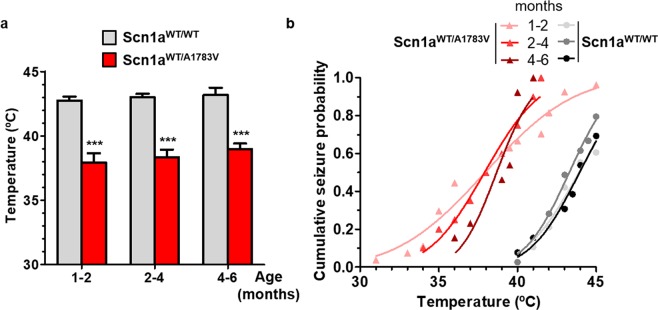
Scn1a^WT/A1783V^ are prone to suffer heat-induced seizures. Mice of the indicated age ranges were exposed to controlled hyperthermia in a chamber with 0.5 °C increments in temperature every 30 s up to 45 °C or until a generalized seizure was reached. (**a**) Thresholds were significantly lower in Scn1a^WT/A1783V^ compared with their control littermates at all age ranges tested. These differences were maintained throughout all the age range tested (Scn1a^WT/WT^: 1–2 mo n = 23, 2–4 mo n = 27 and 4–6 mo n = 9; Scn1a^WT/A1783V^: 1–2 mo n = 26, 2–4 mo n = 20 and 4–6 mo n = 13). ***p < 0.001, One-way ANOVA with Tukey’s post-test. (**b**) Cumulative seizure probability showing that the risk of seizures is confined between 40–45 °C in all ages in Scn1a^WT/WT^ mice, whereas Scn1a^WT/A1783V^ present a wider temperature range at young ages.

In order to define the seizure-threshold temperature of this DS model, Scn1a^WT/A1783V^ and their corresponding control littermates were subjected to hyperthermia at different age intervals (1–2, 2–4 and 4–6 months of age). All Scn1a^WT/A1783V^ mice suffered clinically recognizable seizures, with an average threshold of 38.2 ± 2.9 °C (Fig. 3a). In contrast, 80% of Scn1a^WT/WT^ mice experienced seizures only when temperatures increased to a maximum of 45 °C (Fig. 3b), showing an average threshold of 42.9 ± 1.4 °C.

**Figure 4:**
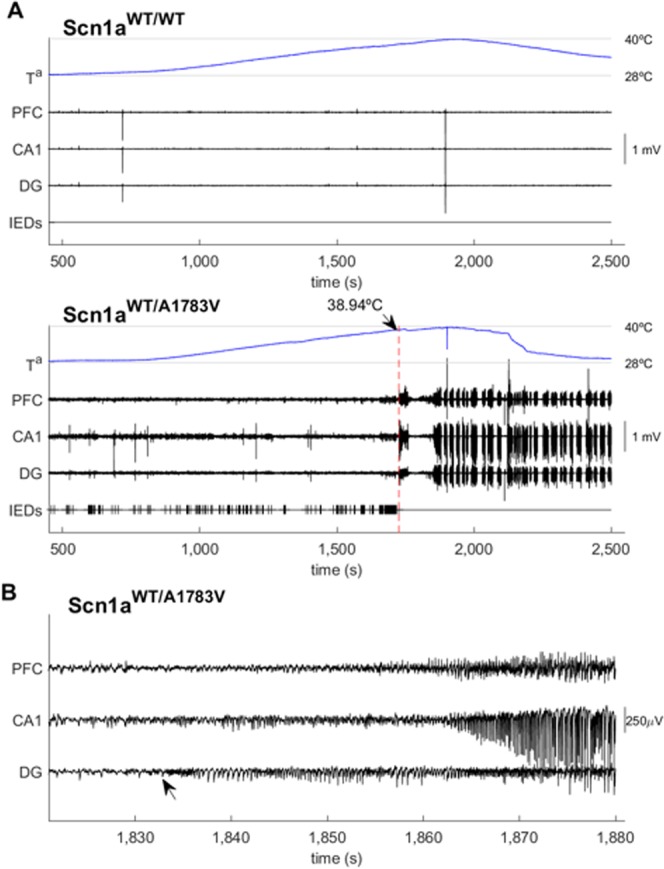
Electrophysiological characterization of freely moving Scn1a^WT/A1783V^ mice. (**a**) Example of the electrical activity recorded in a Scn1a^WT/WT^ (top) and Scn1a^WT/A1783V^ (bottom) mouse during the thermal challenge. Two months-old animals (n = 5) were introduced in the heating chamber, and temperature was increased gradually while electrical activity was recorded in the prefrontal cortex/frontal associative cortex (PFC), CA1 and dentate gyrus (DG) regions. The heat source was switched on at t = 800 s. In the case of the Scn1a^WT/A1783V^ mouse the seizure starts around t = 1,700 s, at T = 38.94 °C. Despite the heat source was immediately disconnected, seizures persisted and were organized in clusters, even when the animal was removed from the recording chamber (around t = 2,250 s). Note the presence of IEDs at RT (t < 1,000 s) and how they increase in frequency as the temperature rises, reaching a maximum right before the seizure (bottom). In contrast, neither IEDs nor seizures were observed in the case of the Scn1a^WT/WT^ mouse (top). (**b**) Example of a seizure with focal origin in the DG that is further generalized (see arrow).

Multisite recordings (hippocampus and prefrontal cortex) were performed in a subgroup of animals from the 1–3 age range to investigate electrophysiological differences between Scn1a^WT/A1783V^ and Scn1a^WT/WT^ littermates (Fig. 4). Thirty minutes of electrophysiological activity together with synchronized digital video recording in awake, freely moving mice in an open-field arena revealed the presence of interictal epileptiform discharges (IEDs) in all of the Scn1a^WT/A1783V^ mice (5 out of 5) at room temperature (RT). IEDs discharges consisted in isolated or grouped spikes (*multispike*) with very short durations (<20 ms) and high amplitudes (>100 µVpp) standing above the background activity (Fig. 4a). Both, focal (i.e. presence of IEDs in a single channel) and generalized (presence of IEDs across several channels simultaneously) distributions were observed. Interestingly, no IEDs were detected in any of their Scn1a^WT/WT^ littermates (5 out of 5). Mice’s brain activity was then recorded at increasing temperatures (Fig. 4a). None of the Scn1a^WT/WT^ mice showed evidence of clinic (electrical seizures with behavioral manifestations) or subclinic (electrical seizures without clear behavioral manifestation) seizures nor the presence IEDs within the range of temperatures investigated (28–42 °C). On the contrary, Scn1a^WT/A1783V^ mice showed an increase in the rate of IEDs at increasing temperatures (Fig. 4a) that ultimately led to the appearance of clinical (4/5) or subclinical (1/5) heat-induced seizures in all the animals. Seizures in Scn1a^WT/A1783V^ mice persisted for several minutes. Visual inspection of the simultaneous video and electrophysiological recordings allowed correlation between changes in the electrophysiological activity and behavioral manifestations following a revised Racine scale (rRS) for mice^25^. At RT IEDs appeared randomly superimposed on the ongoing baseline activity with no behavioral manifestation (no jerks, whisker trembling nor behavioral arrest). At increasing temperatures, behavioral manifestation compatible with scores 0–2 of the rRS where accompanied with electrical activity deceleration and in some cases, increases in the rate of IEDs. When approaching to the onset, electrical activity became more regular (rhythmic) with slightly smaller amplitude and behavioral manifestations included neck jerks, head nodding and clonic tail elevation (score 3) together with falls of the animal into a sitting position or presence of tonic or clonic contractions in one of the legs (compatible with scores 4–5 of the rRS). Then, electrical activity suddenly changed and showed intermittent clusters of high amplitude polyspikes and spike-wave discharges separeated by periods where EEG traces appeared almost flat (although there was still some electric activity). High amplitude polyspikes and spike-wave periods were accompanied by behavioral manifestations compatible with rRS 5 −6 (i.e clonic and tonic-clonic seizures lying on belly/side or wild jumping). Silent periods were accompanied by episodes of arrest with tonic extension of the muscles corresponding to the maximum score (7). In some cases, seizures persisted after removing the animal from the recording/heating chamber and stopped only when placing mice in a colder environment. In one case, the increase of temperature induced a status epilepticus (SE) of generalized tonic-clonic seizures leading to death.

In two cases electrophysiological recordings allowed detection of a focal origin in the dentate gyrus of the hippocampus with further generalization to other areas of the hippocampus and prefrontal cortex (see Fig. 4b).

### Scn1a^WT/A1783V^ mice show cognitive impairment

**Figure 5:**
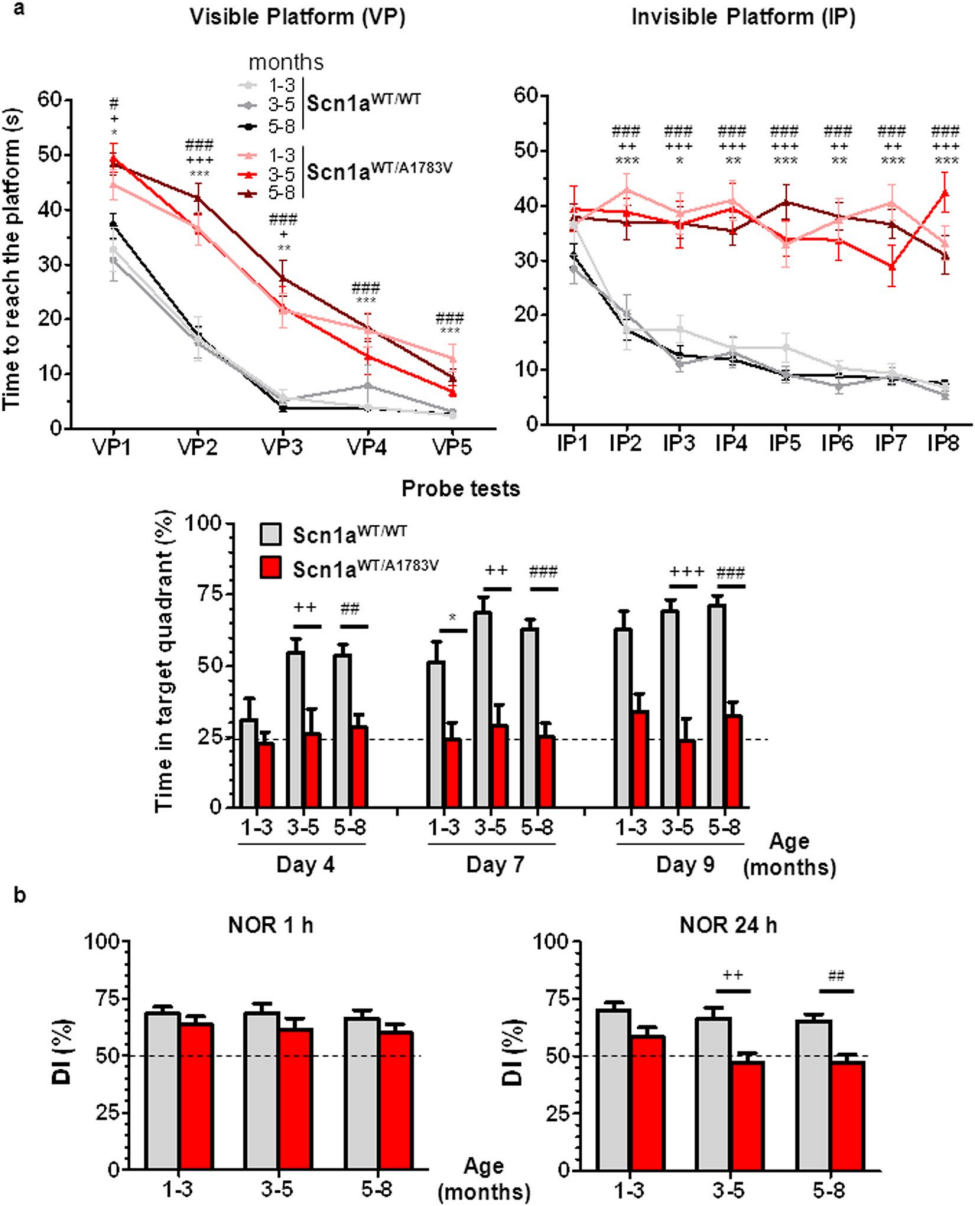
Scn1a^WT/A1783V^ mice show cognitive alterations involving task learning and visuospatial memory. (**a**) The MWM test performed at different ages showed increased escape latency in the visible platform (VP) in the Scn1a^WT/A1783V^ mice compared with their littermate controls, although significant learning was present in both groups of mice (p < 0.001 Friedman test). In contrast, the performance in the invisible platform (IP) showed differences both in absolute latencies and the slope of curves, indicating that Scn1a^WT/A1783V^ mice are unable to improve their escape latency throughout the training (p > 0.05 and p < 0.001 for Scn1a^WT/A1783V^ and Scn1a^WT/WT^ mice, respectively, Friedman). In concordance with the lack of spatial learning, Scn1a^WT/A1783V^ mice showed no preference for the target quadrant in the probe test, consistent with altered retention. Values are represented as mean ± SEM (Scn1a^WT/WT^: 1–3 mo n = 11, 3–5 mo n = 12 and 5–8 mo n = 27; and Scn1a^WT/A1783V^: 1–3 mo n = 17, 3–5 mo n = 10 and 5–8 mo n = 24). (**b**) The NOR test was applied to mice in the same age groups. Although no differences were observed 1 h after training (left panel), a significant reduction in long-term memory was noted in Scn1a^WT/A1783V^ mice (NOR 24 h), revealing a defect in memory consolidation. Values are represented as mean ± SEM (Scn1a^WT/WT^: 1–3 mo n = 37, 3–5 mo n = 26 and 5–8 mo n = 27; Scn1a^WT/A1783V^: 1–3 mo n = 28, 3–5 mo n = 30 and 5–8 mo n = 37). Statistical relevance was assessed applying one-way ANOVA with Tukey’s post-test for VP1–2, IP5, IP8, Probe 15 day 4, Probe 15 day 7, NOR 1 h and NOR 24 h or Kruskal-Wallis with Dunn’s post-test for VP3–5, IP1–4, IP6–7 and Probe 15 day 9. *p < 0.05, **p < 0.01, ***p < 0.001 for comparison of Scn1a^WT/A1783V^ and Scn1a^WT/WT^ mice. Symbols *, ^+^ and ^#^ correspond to the 1–3, 3–5 and 5–8 months age ranges, respectively.

Spatial learning and memory were evaluated using the Morris water maze (MWM) test at different age intervals (1–3, 3–5 and 5–8 months). The visible platform phase of the test revealed that Scn1a^WT/A1783V^ mice learned new tasks more slowly than their control littermates, which is consistent with the cognitive delay observed in DS patients (Fig. 5a). The difference between both groups was more dramatic in terms of spatial memory, as shown in the invisible platform phase of the test. This indicates that Scn1a^WT/A1783V^ mice are unable to use visual cues to accelerate the location of the hidden platform. In agreement with a defect in retention, the probe test showed significant differences in all age groups.

The novel object recognition tests revealed no differences in short-term visuospatial memory (1 h after training) (NOR 1 h, Fig. 5b). However, long-term visuospatial recognition memory impairment was observed in Scn1a^WT/A1783V^ mice compared to control littermates at mild and late stages of the disease (NOR 24 h, Fig. 5b), indicating a fail of memory consolidation in this animal model that supports the results obtained in the MWM.

### Scn1a^WT/A1783V^ mice suffer motor alterations

**Figure 6:**
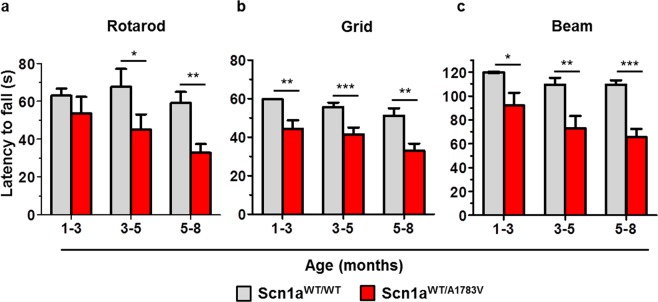
Scn1A^WT/A1783V^ mice show motor impairment. (**a**–**c**) Mice of the indicated age ranges were subjected to the rotarod, inverted grid and elevated beam tests, as indicated. The latency to fall from the rotatory rod was decreased in the Scn1A^WT/A1783V^ mice compared with their control littermates, with significant differences observed from 3 months of age. For the other two tests, a significant reduction was observed in all age groups. Each bar represents the mean ± SEM of elapsed time (s) and are the mean of at least two trials (Scn1a^WT/WT^: 1–3 mo n = 31/25/19, 3–5 mo n = 16/19/14 and 5–8 mo n = 24/14/26; Scn1a^WT/A1783V^: 1–3 mo n = 18/13/11, 3–5 mo n = 16/29/14 and 5–8 mo n = 25/16/31; respectively in each of the tests performed). *p < 0.05, **p < 0.01, and ***p < 0.001. One-way ANOVA with Tukey’s post-test for (**a**) and Kruskal-Wallis with Dunn’s post-test for (**b**,**c**).

Gait and movement abnormalities were apparent in Scn1a^WT/A1783V^ mice right after weaning. In order to quantify motor skills, mice were subjected to a battery of tests, including rotarod, inverted grid and elevated beam. Altogether, these tests revealed a significant impairment in coordination and balance, compatible with the clinical observations in DS patients (Fig. 6). In general, motor performance declined over time in Scn1a^WT/A1783V^ mice, although this trend only reached statistical significance in the rotarod test when the early and late age groups were compared (Fig. 6a).

### Scn1a^WT/A1783V^ mice present hyperactivity and anxiety

**Figure 7:**
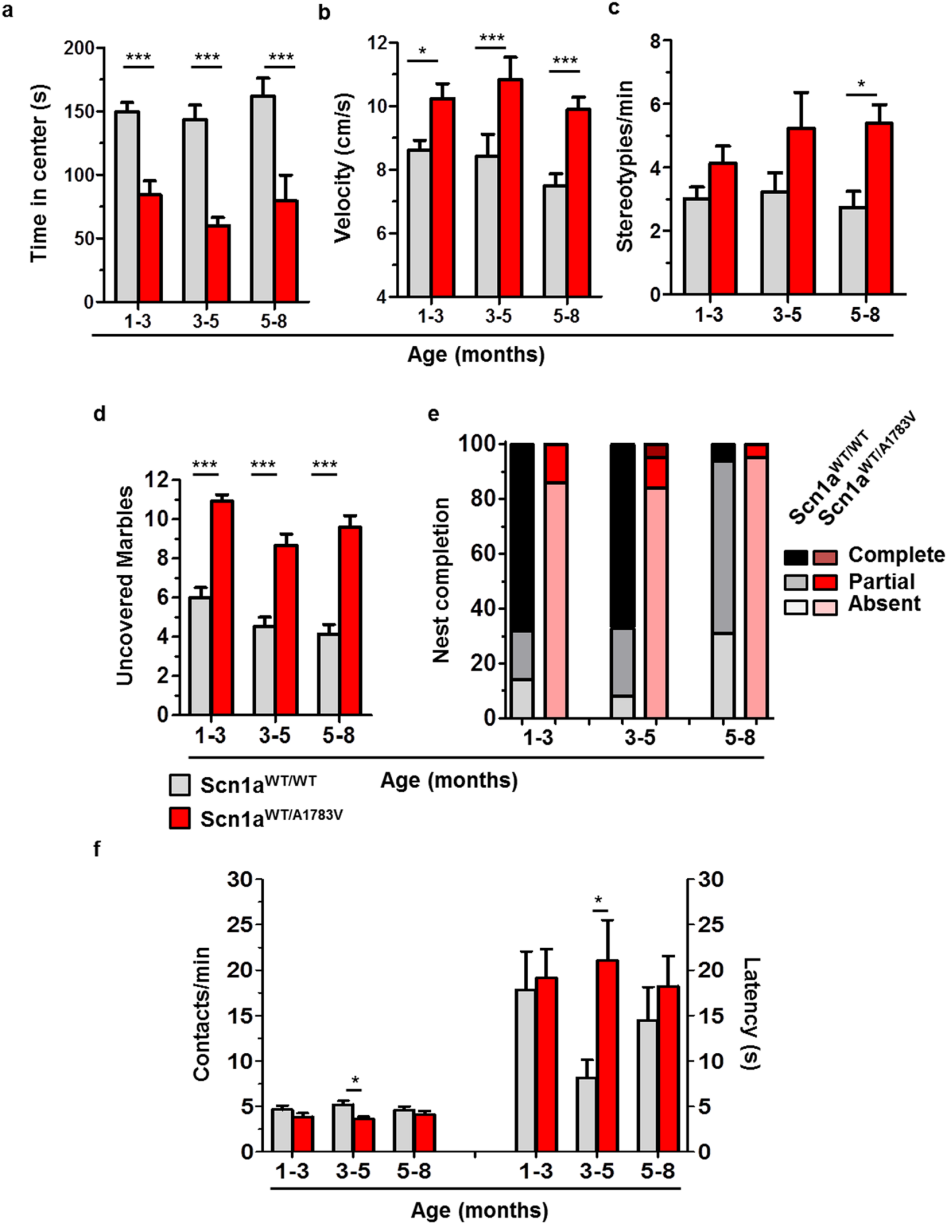
Scn1A^WT/A1783V^ mice show an altered interaction with the environment. Mice of the indicated age ranges were subjected to the open-field test and showed a reduction in the time spent in the center of the arena -indicative of anxiety- (**a**), hyperkinesia (**b**) and an increased number of stereotypies (**c**). No differences in total distance moved were detected in any age-range (data not shown). Other alterations of animal behavior included a reduction in the normal tendency to hide objects in the marble burying test (**d**) and a poor performance in the nest building test (**e**). The graph represents the percentage of mice that complete, initiate or fail to initiate the task during one night (dark, medium and light colors, respectively). In contrast, Scn1A^WT/A1783V^ performed relatively well in the social interaction task (**f**), with a reduction in the number of contacts only observed in the 3–5 months age range (left Y axis), which coincided with a significantly higher latency to approach the unfamiliar mouse for the first time (right Y axis), compared with their control littermates. Values are represented as mean ± SEM (Scn1a^WT/WT^: 1–3 mo n = 44/44/14/26/16/16, 3–5 mo n = 31/30/13/34/6/11, and 5–8 mo n = 29/27/17/8/17/18; Scn1a^WT/A1783V^: 1–3 mo n = 26/29/14/20/6/15, 3–5 mo n = 33/34/9/37/12/11 and 5–8 mo n = 35/36/24/18/20/15; respectively in each of the test performed). *p < 0.05; ***p < 0.001, Kruskal-Wallis with Dunn’s post-test.

Behavioral manifestations in DS mouse models are heavily influenced by the specific *Scn1a* defects and the genetic background^18–20^. In order to investigate this aspect in Scn1a^WT/A1783V^ mice we employed the open-field test. Whereas total distance moved was not different from their control littermates (not shown) the most dramatic difference observed was the time spent in the center of the arena (Fig. 7a). This is a strong indicator of anxiety behavior in these mice. In addition, Scn1a^WT/A1783V^ showed faster peak velocities than controls (Fig. 7b), suggesting hyperactivity/impulsivity. This result is in agreement with the behavior observed during animal handling. In support to the hyperactive behavior, we observed an increase in the number of repetitive movements (stereotypies), starting at 3 months of age (Fig. 7c). In parallel with these signs of hyperactivity, exploratory behavior was severely impaired, as revealed by the marble burying test. Scn1a^WT/A1783V^ mice showed a marked reduction in the normal tendency of rodents to cover objects found in their cages (Fig. 7d). With the aim of studying a complex, species-adapted behavior that could evaluate the individual’s performance in daily life activities, we carried out the nest building test. A dramatic reduction in nest assembly was observed in Scn1a^WT/A1783V^ mice, with more than 80% of individuals unable to initiate building at all age intervals tested (Fig. 7e). In contrast with these behavioral alterations, we could only find mild signs of impaired sociability when mice were subjected to the social interaction test^26^. Scn1a^WT/A1783V^ mice in the intermediate age group showed a moderate reduction in contacts during the 15 min period of co-habitation, which was concomitant to an increase in the latency to first contact (Fig. 7f). Therefore, it seems that the main reason for the reduced number of contacts is the delay in the establishment of the first interaction, which could be influenced by the other motor and behavioral defects. This finding is in line with the clinical features of DS patients, whose social abilities are more preserved than the communication skills due to their cognitive impairment^27^.

### Scn1a^WT/A1783V^ mice show increased glucose uptake in the brain

**Figure 8:**
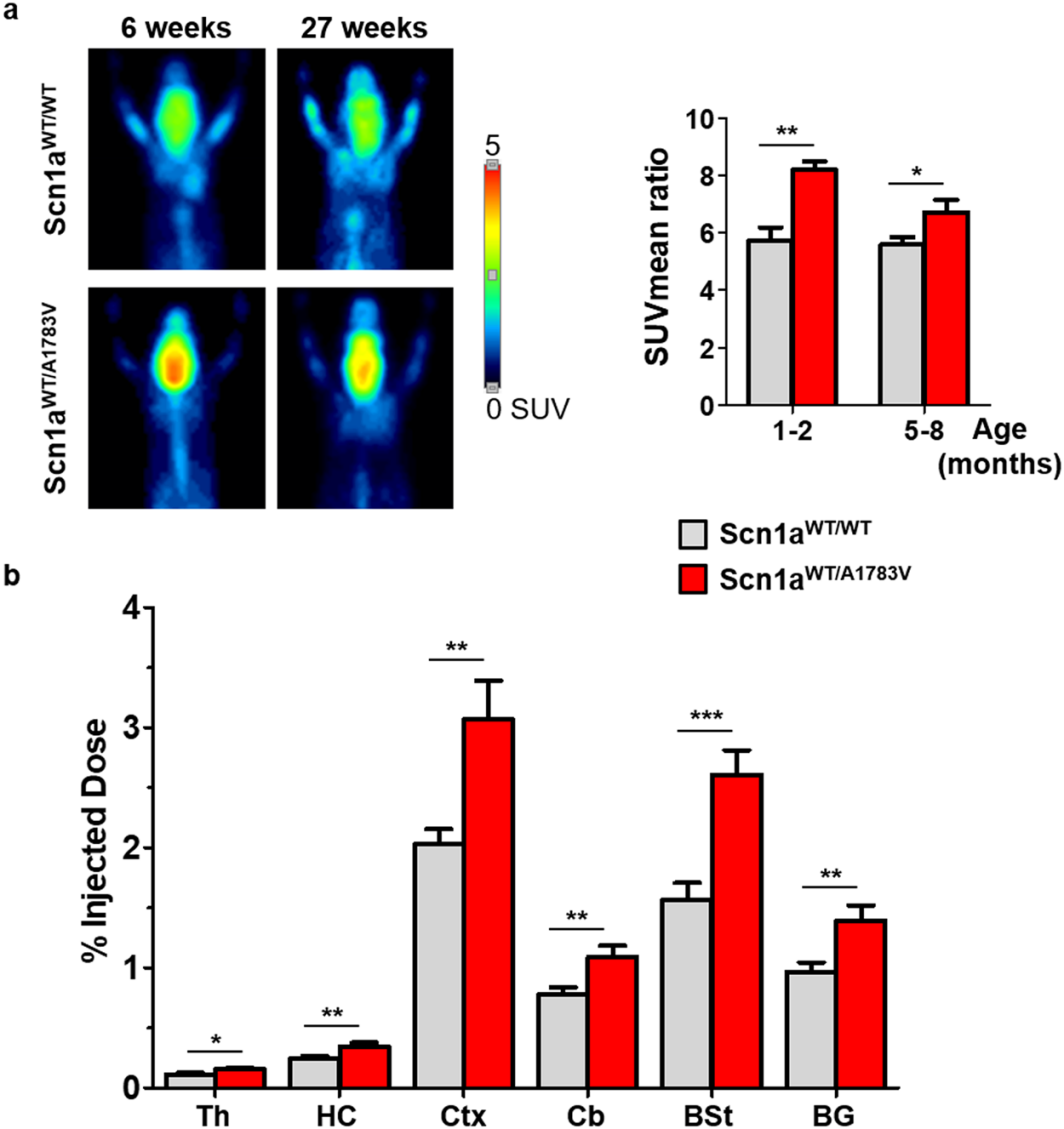
Scn1a^WT/A1783V^ mice show increased glucose uptake in the brain. Mice were subjected to ^18^F-FDG PET at the indicated ages. (**a**) Left panel correspond to maximum intensity projection PET images of representative mice showing brain ^18^F-FDG uptake. The quantification of positron emission (mean SUV) is represented in the right panel. (**b**) Mice in the 5–8 age group were sacrificed after PET, and isotope incorporation was quantified in a gamma counter (expressed as % of the injected dose). (1–2 mo n = 7; 5–8 mo n = 14). *p < 0.05; **p < 0.01. Mann Whitney U test.

It has been recently described that DS patients develop abnormal brain glucose uptake starting at 6 years of age^28,29^. In particular, reduced glucose uptake was reported in the fronto-temporo-parietal cortices. Although studies are still very limited, this indicator of cellular metabolism could become a prognostic factor for brain function and/or a marker for therapeutic response in these patients. Therefore, we performed^18^ FDG-PET in young and middle-aged Scn1a^WT/A1783V^ mice and age-matched controls that were not subjected to any thermally-induced seizure. In contrast with current clinical reports, we observed a global increase in glucose uptake specifically in the brain of Scn1a^WT/A1783V^ mice. A net increase in brain emission was demonstrated, using the liver as a reference organ (Fig. 8a). Mice were then sacrificed and different brain structures were isolated for quantification of radionuclide incorporation. Higher uptake was observed in all regions, including cerebellum, basal ganglia, brainstem, cortex, thalamus and hippocampus (Fig. 8b). Potential reasons for the apparent discrepancy between recent clinical reports and our results are discussed below.

## Discussion

DS is a complex encephalopathy affecting the inhibition/excitation balance in the brain, which explains the wide repertoire of clinical manifestations. Apart from the epileptic seizures, other comorbidities have a deep impact on the quality of life of the patients and their families. Therefore, addressing SUDEP, motor, cognitive and behavioral alterations are clear objectives for new experimental therapies looking for patient-centered outcomes^30^. In this context, relevant pre-clinical models and robust methods to quantify response to treatments are becoming a priority in the field. In this report we have characterized a mouse model in which a missense *Scn1a* mutation recurrently observed in patients is present in all cells, thus mimicking a clinically relevant situation at the genetic level. Our results indicate that the most relevant DS manifestations are present in Scn1a^WT/A1783V^ mice, and can be readily quantified using standardized tests. These include a marked sensitivity for thermally-induced seizures, EEG alterations, progressive motor impairment, hyperactivity and cognitive deterioration.

Behavioral alterations are probably the most variable manifestations among DS patients, and the same occurs in pre-clinical mouse models. Apart from the inherent difficulty to discriminate different behavior traits using rodent tests, there is a strong influence of the genetic background and the type of cells affected by the *Scn1a* alterations. In the model described here, the open-field test was a robust method to quantify anxiety (time spent in the center of the arena). Although the high frequency of stereotypies observed in the Scn1a^WT/A1783V^ mice could be considered an autistic-like behavior, we believe that in this case it is mainly a reflection of hyperactivity. This is in agreement with the increased velocity in the open-field and the hyper-reactivity experienced during animal handling. We believe the striking reduction in nest building performance observed in Scn1a^WT/A1783V^ mice is a relevant indicator of poor quality of life and a clear parameter to be considered in the evaluation of new therapies. In contrast, evidences for reduced sociability were not consistent, according to the interaction test. This is probably influenced by the C57BL/6 J genetic background, which is relatively resistant to social interaction defects^20,31^. In fact, PV-specific happloinsufficiency of *Scn1a* in this background showed normal social behavior^20^, and only the global introduction of a nonsense mutation (Scn1a^RX/+^ mice) caused alterations in sociability tests. On the other hand, reduced social skills in DS patients are better explained by their difficulties in communication and their cognitive impairment rather than by specific deficits in socialization^27^. In addition, the contribution of cerebellar alterations to an internalizing syndrome should be taken into account^32,33^.

In comparison with the previously described knock-in model carrying the R1648H mutation associated with GEFS^+^ (*Scn1a*^RH+^ mice)^34^, Scn1a^WT/A1783V^ have in common the hyperactivity and mild social deficits, whereas the DS mutation causes deeper cognitive impairment, anxiety and higher mortality. Interestingly, a recent study indicates that the phenotype of heterozygous *Scn1a*^RH+^ mice can be aggravated by repeated hyperthermia-induced crisis, resembling DS^35^. This finding highlights the interplay between genetic and environmental factors in disease progression.

Finally, we report for the first time an FDG-PET study in a DS murine model, which could be an indirect, but clinically relevant marker for brain function in this disease. In contrast with the pioneering reports in DS patients^28,29^, we found an increase in glucose uptake in different brain structures. This is not surprising, taking into account the unbalanced excitatory activity resulting from the insufficient *Scn1a* function. We believe the apparent reduction of glucose uptake observed in the clinical studies can be influenced by the anti-epileptic drugs used in patients^36^, and by the normalization method used in these studies. Haginoya *et al*. took cerebellum as a reference^28^, whereas Kumar *et al*. used basal ganglia^29^. Our data indicate that both brain regions have elevated uptake positron emission, which complicates the interpretation of results. Brain metabolism is generally reduced in drug-resistant epilepsies^37^, especially as a result of prolonged exposure to seizures. However, hypermetabolism has been described in interictal periods when continuous epileptogenic activity is present^38^.

To conclude, we present here an “*open access*” DS mouse model based on a clinically relevant genetic alteration, which allows the quantitative evaluation of a wide repertoire of disease manifestations. The selection of methods described in this report cover the most relevant parameters at the clinical, electrophysiological and metabolic levels. We believe this may be a useful tool to test novel therapies and to obtain meaningful data across different research groups.

## Materials and Methods

### Animals

The conditional *Scn1a*-A1783V mice (B6(Cg)-Scn1atm1.1Dsf/J, The Jackson Laboratory, stock no. 026133) were bred to mice expressing Cre recombinase under the control of the CMV promoter (B6.C-Tg(CMV-Cre)1Cgn/J, The Jackson Laboratory, stock no. 006054^39^). Breeding pairs consisted of heterozygous male *Scn1a*-A1783V and homozygous female CMV-Cre mice. See https://www.jax.org/strain/026133 for details about allele modification and genotyping. Offspring carrying one mutated allele (genotype hereinafter referred to as Scn1a^WT/A1783V^) express the A1783V mutation in the *Scn1a* gene in all body tissues, mimicking what happens in DS. Animals were housed 4–6 per cage with free access to food and water, weighed weekly, and maintained in a temperature and light controlled (12 h/12 h light/dark cycle) environment. The studies were performed by comparing heterozygous transgenic Scn1a^WT/A1783V^ to age-matched negative littermates Scn1a^WT/WT^. Breeding and experimental protocols were approved by the Ethical Committee of the University of Navarra (in accord with the Spanish Royal Decree 53/2013).

### Quantitative PCR

In order to minimize the number of animals employed in this part of the study (limited to 6 Scn1a^WT/WT^ and 8 Scn1a^WT/A1783V^), one brain hemisphere and half cerebellum of each animal were dissected and employed to determine mRNA levels of the *Scn1a* gene by quantitative PCR, and the other one for the determination of Nav1.1 protein levels by Western blotting. After dissecting the cerebral cortex, hippocampus and cerebellum, the Maxwell^®^ 16 LEV simplyRNA Cells Kit (Promega, Madison, WI, USA) was used for total RNA isolation following manufacture’s indications. Two micrograms of RNA were then treated with DNase I and retro-transcribed into cDNA using M-MLV retro-trasncriptase enzyme (Invitrogen, Thermo Fisher Scientific, Carlsbad, CA, USA) and random primers (Life Technologies, Thermo Fisher Scientific, Carlsbad, CA, USA). These procedures were performed in GeneAmp^®^ PCR System 2400 (Applied Biosystems, Foster City, CA, USA). Quantitative analysis was performed by real-time PCR using iQTM SYBR^®^ Green Supermix reagent (Bio-Rad, Hercules, CA, USA) in CFX96 TouchTM Real-Time PCR Detection System (Bio-Rad, Hercules, CA, USA). Mouse *Scn1a* expression levels were determined using specific primers (FP 5′ CATGTATGCTGCAGTTGATTCCA 3′and RP 5′ AACAGGTTCAGGGTAAAGAAGG 3′)^40^ and mouse GAPDH was used as housekeeping gene (FP 5′ CCAAGGTCATCCATGACAAC 3′and RP 5′ TGTCATACCAGGAAATGAGC 3′). All brain samples were tested in triplicate. The relative quantification was carried out using the 2−∆Ct^41^. Data are analysed in percentage *vs* Scn1a^WT/WT^ mice and represented as mean ± SEM.

### Preparation of membrane-enriched extracts and western blotting

The cerebral cortex and hippocampus of the other hemisphere and half cerebellum were employed to obtain the membrane-enriched protein fraction (P2) as previously described^42^. Protein concentration was determined by Bradford assay (BioRad Laboratories, Hercules, CA) and part of the preparation was solubilized in denaturing conditions as described before^43^. Protein samples were mixed with 4x Urea-EDTA buffer, resolved onto SDS-polyacrylamide gels and transferred to PVDF membranes. The membranes were blocked with 5% BSA and 0.05% Tween-20 in TBS, followed by overnight incubation at 4 °C with the following primary antibodies: rabbit polyclonal anti-Nav1.1 (Alomone Labs Cat# ASC–001, Cat# 2118S, 1:500) and rabbit moclonal (14C12) anti-GAPDH (Cell Signaling Technology, 1:5,000) diluted in 2,5% BSA, 0.05% Tween–20 and 0,01% azide in TBS. Immunolabeled protein bands were detected by using and anti-rabbit IgG HRP conjugate (GE Healthcare, Cat# NA934V, 1:10,000) and an enhanced chemiluminiscence system (Lumigen ECL Ultra TMA–6, Lumigen, Inc., Cat# TLA–100). Images were acquired with a Chemidoc system (Biorad, Hercules, CA, USA), and Image Lab^TM^ software (Biorad, Hercules, CA, USA) was used for quantification. The employed anti-Nav1.1 antibody is an affinity-purified rabbit polyclonal antisera raised against synthetic peptides corresponding to the intracellular loop between domains I and II of rat Nav 1.1. It has been KO validated^44^ and validated for Western blotting immunofluorescence in several publications^45–48^ Data are analysed in percentage *vs* Scn1a^WT/WT^ mice and represented as mean ± SEM.

### Immunofluorescence procedures, equipment and settings

Additional animals were perfused transcardially with 0.9% saline followed by 4% paraformaldehyde in phosphate buffer (PB) under xylazine/ketamine anesthesia. After perfusion, brains were removed and post-fixed in the same fixative solution for 12 h at 4 °C; and then cryopreserved in 30% sucrose solution in PB at 4 °C until they sank. Microtome sections (thickness: 30 μm) were cut sagitally with a freezing microtome and stored in cryopreserving solution (30% ethylene glycol, 30% glycerol in PB 0.1 M) at −20 °C until processed. To carry out the immunofluorescence, two free-floating tissue sections per animal were processed (n = 4 in each group). Brain sections were washed 3 times with PB at RT and then a blocking step was performed, followed by overnight incubation at 4 °C with the primary antibody (rabbit polyclonal anti-Nav1.1, Alomone Labs Cat# ASC–001, 1:500) diluted in blocking solution (2% donkey normal serum, 0.5% Triton X–100 and 1% BSA in PB). After washing them 3 times with PB, slices were incubated with the secondary antibody (Donkey anti-Rabbit IgG (H + L) Highly Cross-Adsorbed Alexa Fluor 488 Cat# A–21206, 1:400) for 2 h at RT and protected from light. To enable the visualization of nuclei, sections were incubated for 5 min with the DNA marker 4′,6–diamidino–2–phenylindole (DAPI, ThermoFisher Scientific Cat# D1306, 300 nM) protected from light. Finally, slices were washed twice with PB, mounted on super frost plus slides, air dried for 24 h, rinsed in toluene (2 × 5 min), and coverslip was placed with Immu-Mount^®^ mounting medium (ThermoFisher Scientific Cat# 9990402). To ensure comparable immunostaining, sections were processed together under identical conditions. For the assessment of non-specific primary and secondary immunostaining, some sections from each experimental group were incubated without primary or secondary antibody, and no immunostaining was observed in any case. Fluorescence signals displayed in Fig. 2c were acquired with the fluorescence microscope Eclipse Eboom (Nikon) coupled to a super high-pressure mercury lamp (C-SHG1, Nikon, Japan) using a Plan Apo 10 ×/ 0.45 DIC L objective. Images were acquired with the DS-Ri2 camera (Nikon, Japan. Capture quality: 3 × 8–bit, 4908 × 3264) and the program NIS-Elements F 4.60.00 64-bit (Nikon, Japan. Image information: 8 bits, calibration 1,17 µm/px, and dimensions 808 × 808). The B-2A filter (excitation: 450–490 nm, DM: 505, BA: 530) was used to detect Nav1.1–inmunostained structures and the UV-2A one (excitation: 330–380, DM: 400, BA: 420) for DAPI-staining. Emissions were color-coded in green and blue, respectively. Acquired fluorescence images were adjusted in parallel for brightness and contrast in ImageJ 1.52p (NIH, Bethesda, MD), sharpness was improved employing an unsharp mask filter (Radius (sigma): 1.0 px, and mask weight; 0.60). For bigger magnification (Fig. 2d) tissue sections were visualized using a confocal laser scanning microscope (Zeiss Axio Observer.Z1/7 LSM800 with Airyscan and ESID 2XGaAsP detector module) and a Plan-Apochromat 63 ×/ 1.40 Oil DIC M27 objective. Tissue sections were excited using a 488 nm laser for detecting Nav1.1-inmunostained structures (emission wavelength 509 nm, detection wavelength 480–700 nm, pinhole 50 µm) and a 353 nm laser for DAPI-staining (emission wavelength 465 nm, detection wavelength 400–480 nm, pinhole 50 µm). Emissions were color-coded in green and blue, respectively. Images were acquired with the program ZEN 2 (blue edition) (Carl Zeiss. Dimensions: 512 × 512, 16-bit; and image size: 190.16 × 190.16 µm). Acquired fluorescence images were adjusted in parallel for brightness and contrast in ImageJ 1.52p (NIH, Bethesda, MD), noise was removed applying a bright outlier detection filter (Radius: 0.1 px, and threshold: 90) and sharpness was improved employing an unsharp mask filter (Radius (sigma): 1.0 px, and mask weight: 0.20).

### Induction of thermal seizures

In order to evaluate the epileptogenic thermal threshold in our animal model at different ages, a methacrylate cylinder coupled to a thermal system that gradually increased its internal temperature was used. Before introducing the animal into the cylinder, its body temperature was measured employing a rectal probe (RET-4, Physitemp Instruments, LLC) coupled to the TCAT-2LV controller (Physitemp Instruments, LLC). The environmental temperature was gradually increased to a maximum of 45 °C (0.5 °C every 30 s) or until a generalized seizure was reached. After removing the animal from the cylinder and measuring its body temperature as described before, the animal was left in a cool box with free access to water to help it recover.

### Electrophysiological recordings

Electrophysiological phenotyping of the DS model was carried out by multisite recordings in 5 Scn1a^WT/A1783V^ and 5 Scn1a^WT/WT^ mice at 2 months of age (71 ± 18 days). Local field potentials (LFP) from the hippocampus CA1 region and prefrontal cortex together with simultaneous video recordings were obtained. To do that, mice were implanted with 5 equally spaced 50 µm tungsten wires (California Fine Wire, CA 93433, USA) across different layers of CA1 and dentate gyrus (DG) and a wire at prefrontal cortex, frontal associative cortex (PFC). Coordinates for electrode placement were selected according to Paxinos and Watson atlas: (AP): −1.94 mm; (ML): 1.5 mm; (DV): −2 to −1 mm for the hippocampal bundles and (AP): 2.58 mm; (ML): 1.5 mm; (DV): −1.5 mm for the PFC. Reference and ground screws were placed over the cerebellum. One week after implantation (time for recovery from surgery and inflammation, as recommended by the ethical committee), animals were connected to an acquisition system for electrophysiological recordings (Intan RHD2000 system, IntanTech). Simultaneous video recordings were used to assess behavioural state of the animals and the presence of (clinical) seizures. Recording session began with 30 min of awake, freely moving recordings of the mice within their cages at RT. Open-field recording was then followed by a thermal challenge where mice were placed into a heating chamber and recorded at increasing temperatures from RT up to 42 °C or the appearance of seizures. Custom-made routines running under Matlab (Mathworks, Natick, MA, USA) were used to convert electrophysiological data and temperature values from Intan format into Spike2 format (Cambridge Electronic Desing Limited, UK). Reviewing features of the Spike2 software were used to visually inspect the recordings in order to assess the presence of abnormal activities (interictal discharges), electrical seizures and temperature values at the onset of the seizures. Interictal epileptiform discharges (IEDs) were semi-automatically annotated by performing an initial detection following previously described methods^49^ and further validated by visual inspection. To do that, signals were loaded using Matlab scripts, resampled to 1,000 Hz, band-pass filtered in the 60–80 Hz range and rectified. IED events were detected when the filtered envelope was > 3 times above baseline and unfiltered envelope was > 3 times above baseline. Envelope was computed by estimating the rms value within a 200 ms window. Then, candidate events were uploaded into the Spike2 file as a marker channel that was further reviewed and curated by two specialists. Video recordings were also reviewed by two different specialist to detect the presence of seizures and to report their semiologycal description according to a modified Racine score for mice^25^. Location of the electrodes was assessed by histological verification. To do that, animals were anaesthetized (ketamine 75 mg/kg and xylacine 11 mg/kg intraperitoneal) and intracardially perfused with a solution of paraformaldehyde (PAF 4%), dissolved in phosphate buffer saline (PBS 0.1 M, pH 7.4). After perfusion, the brain was taken out and post-fixed during 24 h in PAF. Then it was passed to PBS-sacarose for at least 24 h. Brains were cut in coronal axis using a cryotome. Slices of 30 μm were obtained and processed to assess electrodes location. The slides were stained with thionine and then observed in a microscope to verify the location of the recording electrodes.

### Behavioural assessment

#### Morris water maze test

The Morris water maze is a hippocampus-dependent learning task that serves to test the working and reference memory function. The test was carried out as described before with minor modifications^43^. The water maze consisted of a circular pool of 1,2 m diameter and 0,6 m height (LE820120, PanLab Harvard Apparatus) filled with water tinted with non-toxic white paint and maintained at 20 °C. During the first part of the test, Visible Platform (VP), mice were trained to find the platform in order to escape from the water. During this phase, the platform was raised above the water surface and mice were trained for 5 consecutive days (four trials per day). In the second part of the test, Invisible Platform (IP), the platform was placed in the opposite quadrant and hidden bellow water level, and visible cues were placed in each of the four quadrants of the maze to allow spatial learning. Mice were trained for 8 consecutive days (four trials per day) to generate a spatial map that allows them to find the platform. In both phases, mice were placed into the maze facing toward the wall of the pool in selected locations pseudo-randomly established. Each trial was finished when the mouse reached the platform (escape latency) or after 60 s; when an animal was unable to find the platform, it was gently guided onto it. After each trial, animals remained on the platform for 15 s. On days 4^th^, 7^th^ and 9^th^ of the IP phase all mice were subjected to a probe trial in order to evaluate their retention. Platform was removed from the pool and mice allowed to swim during 60 s in the pool, measuring the time spent in the quadrant where the platform was placed during the IP for the first 15 s (Probe 15). All trials were recorded and analysed with the program WaterMaze3 (Actimetrics, Evanston, IL).

#### Novel object recognition test

Novel object recognition test (NOR) was conducted for the assessment of visuospatial memory. A standard squared four compartment open-field box mildly illuminated was employed (LE800SC, PanLab Harvard Apparatus, 90 × 90 × 40 cm). All trials were recorded and exploration times for each object were analysed manually blindly. Before performing the test, animals were habituated to the box during 15 min, these data were employed to evaluate their motor spontaneous activity as it will be described later. The test consists of three different stages; the first one is the habituation phase. Two identical objects were placed into the box, symmetrically separated from each other, and each mouse was allowed to explore them for 5 min. After a delay of 1 h or 24 h, the mouse was placed again in the cage and exposed for 5 min to one familiar object in the same position and to a novel object placed in a new location (NOR 1 h and NOR 24 h phases). All trials were video recorded and the total time spent exploring each object was measured manually by using a stopwatch. To avoid the presence of olfactory trails, the apparatus and the objects were thoroughly cleaned after each trial. The discrimination index (DI) was calculated as percentage following this equation: (Exploration time of the novel object/Total exploration time) × 100. Consequently, a ratio of 50% reflects equal exploration of the familiar and the novel object, indicating no learning retention, and ratios above 50% are indicative of visuospatial learning retention.

#### Rotarod test

Motor coordination, balance and physical condition were tested in a rotarod apparatus (LE8200 Panlab, Harvard Apparatus). The day before the test, animals were trained to walk over the rotating rod for 5 min at a constant speed of 12 rpm. For the test, animals were positioned on a rod programmed to rotate with lineal increasing speed going from 4 to 40 rpm in 5 min. Animals underwent three trials with a resting time of 60 min between them. The time spent in the accelerating cylinder was recorded, representing the mean value. Of note, mice were not subjected to the rotarod test until they weighted at least 10 g, since animals with lower weights showed inconsistent results.

#### Inverted grid test

Grip strength was tested by evaluating the capability of the mouse to remain clinging to an inverted cage lid for 1 min. Animals underwent three trials with a resting time of 60 min between them. The time spent hanging from the lid was recorded, and the score was estimated by the mean value of the three trials.

#### Elevated beam test

In order to assess motor coordination the elevated beam test was used. The test was carried out using a cylindrical, narrow and elevated footbridge. The animal must walk on it for a maximum of two minutes and the time of permanence was evaluated. Animals underwent two trials with a resting time of 60 min between them. The time spent over the footbridge was recorded, and the score was estimated by the mean value of both trials.

#### Open-field test

Motor activity was tested for 15 min in a standardized squared four-compartment open-field box mildly illuminated (LE800SC, PanLab Harvard Apparatus, 90 × 90 × 40 cm). Trials were video recorded and automatically analysed using a video tracking system (Ethovision XT 5.0, Noldus Information Technology B.V., Wageningen, The Netherlands). Parameters as distance moved (cm), mean speed (cm/s) and time spent in the central zone (15 cm apart from the walls) were measured and represented. The last value was employed to evaluate the presence of anxious behaviour. Of note, mice showing very low exploratory activity (less than 5% of the time dedicated to exploration) were excluded from the analysis, since they tend to remain in one corner of the cage and would introduce a bias in the evaluation of anxiety. The presence of stereotypies, indicative of anxiety and hyperactivity, was also evaluated by visual inspection and manually counted.

#### Marble burying test

Normal exploratory behaviour was assessed with the marble burying test^50^. Twelve glass marbles were put uniformly in a cage, three marbles per line, and mice were placed in the centre of each cage and allowed to interact with them for 30 min. After this period of time, two blind experimenters quantified the number of unburied marbles.

#### Nest building test

This test is useful for identifying abnormal behaviour in mice^51^. Animals were placed in a box provided with a piece of tightly packed cotton material (Nestlets™ Nesting Material, Ancare) and let to interact with it overnight. The day after, nesting index was evaluated and scored as no nest, partial nest or complete nest covering the mouse.

#### Social interaction task

In each trial, four pairs of mice were tested simultaneously in a standardized squared four-compartment open-field box mildly illuminated (LE800SC, PanLab Harvard Apparatus, 90 × 90 × 40 cm). Each mouse was introduced in a compartment occupied by a mouse that had never interacted with it (occupant mouse). Both animals could freely interact for 15 min. Trials were video recorded, and the number of social interactions and latency time for the first contact was then manually analysed for each tested mouse.

### Measurement of cerebral glucose uptake by positron emission tomography

Cerebral glucose metabolism, reflecting neuronal and synaptic activity, was assessed *in vivo* by positron emission tomography (PET) with the radiotracer 18– fluorodeoxyglucose (^18^F-FDG). Mice were fasted overnight but allowed to drink water ad libitum. Mice were injected with ^18^F-FDG dose (9,5 MBq ± 0,6 in 80–100 μL) through the tail vein and placed back in the cage for an uptake period of 50 min. Then, animals were anesthetized with 2% isoflurane in 100% O_2_ gas and placed prone to acquire a static 15-min study in a small animal PET tomograph (Mosaic, Philips). Images were reconstructed applying dead time, decay, and random and scattering corrections. For the *ex vivo* studies of radiotracer incorporation, animals were sacrificed at the end of the study by neck dislocation and different parts of brain were dissected. *Ex vivo* counting of radioactivity in the samples was performed in a gamma counter (Hidex Automatic Gamma Counter, Hidex Oy, Turuk, Finland) to calculate the percentage of injected dose (%ID). For the semi-cuantiative analysis of the *in vivo* PET images, studies were analyzed using the PMOD software (PMOD v3.2, PMOD Technologies Ltd., Adliswil, Switzerland). Images were expressed in standardized uptake value (SUV) units, using the formula SUV = [tissue activity concentration (Bq/cm^3^)/injected dose (Bq)] × body weight (g). To assess brain uptake of ^18^F-FDG, two spherical volumes of interest (VOIs) were drawn for each image including the entire brain and liver (reference organ). Then, a semiautomatic delineation tool was used applying a predefined threshold of 50% of the maximum or minimum voxel value to obtain new VOIs that delimited the brain and liver respectively. Finally, the average SUV of the voxels within the VOIs were calculated (SUV mean) and a SUVmean ratio was calculated dividing brain SUV_mean_/ liver SUV_mean_ (SUV_mean_ ratio).

### Statistical analysis

Data were processed for statistical analysis using the Graphpad Prism software. After identifying outliers by applying the Grubb’s test, data normality was assessed with the D’Agostino and Pearson omnibus normality test. In Fig. 1 linear regression analysis was performed to assess statistical significance. In the rest of figures, if data followed normality a one-way analysis of variance (ANOVA) followed by Tukey’s multiple post hoc test was applied; otherwise, Mann Whitney U test or Kruskal-Wallis test followed by Dunn’s test were used. In the WMM, the Friedman test was applied to test intra-group improvement over trials. The significance level was set at p < 0.05.

## Supplementary information

Full-lenght western blots

## Data Availability

The datasets generated during and/or analysed during the current study are available from the corresponding author on reasonable request.
