# Author Correction: Epilepsy and neuropsychiatric comorbidities in mice carrying a recurrent Dravet syndrome *SCN1A* missense mutation

**DOI:** 10.1038/s41598-021-87092-3

**Published:** 2021-04-13

**Authors:** Ana Ricobaraza, Lucia Mora-Jimenez, Elena Puerta, Rocio Sanchez-Carpintero, Ana Mingorance, Julio Artieda, Maria Jesus Nicolas, Guillermo Besne, Maria Bunuales, Manuela Gonzalez-Aparicio, Noemi Sola-Sevilla, Miguel Valencia, Ruben Hernandez-Alcoceba

**Affiliations:** 1 Gene Therapy Program CIMA, IdiSNA, Navarra institute for health research, University of Navarra, Pamplona, Spain; 2 Department of Pharmacology and Toxicology, IdiSNA, Navarra institute for health research, University of Navarra, Pamplona, Spain; 3 Dravet Syndrome Unit, Pediatric Neurology Unit, IdiSNA, Navarra institute for health research, University Clinic of Navarra, Pamplona, Spain; 4 Dracaena Consulting, Madrid, Spain; 5 University of Navarra, Neuroscience Program CIMA, IdiSNA, Navarra institute for health research, Neurophysiology Service, Clinica Universidad de Navarra, University of Navarra, Pamplona, Spain; 6 Neuroscience Program CIMA, IdiSNA, Navarra institute for health research, University of Navarra, Pamplona, Spain

Correction to: *Scientific Reports* 10.1038/s41598-019-50627-w, published online 02 October 2019

The original version of this Article contained errors.

In the Introduction, the identification of the described mouse model was incorrect. In addition, a hyperlink to external data was broken. The correct hyperlink is https://dravet.eu/novel-open-access-mouse-model-of-dravet-syndrome/.

As a result,

“Of note, a knock-in model harboring the same mutation (B6(Cg)-Scn1atm1.1Dsf/J strain crossed with Cox2-Cre expressing mice) has been recently adopted by the US National Institute of Neurological disorders and Stroke (NINDS) in the panel of animal models of the Epilepsy Therapy Screening Program (ETSP). This is the first model of genetic epilepsy to be included in the panel (https://dravet.eu/projects-item/mouse-model/).”

now reads:

“Of note, a knock-in model harboring the same mutation (B6(Cg)-Scn1atm1.1Dsf/J strain crossed with Sox2-Cre expressing mice) has been recently adopted by the US National Institute of Neurological disorders and Stroke (NINDS) in the panel of animal models of the Epilepsy Therapy Screening Program (ETSP). This is the first model of genetic epilepsy to be included in the panel (https://dravet.eu/novel-open-access-mouse-model-of-dravet-syndrome/).”

The original Article has been corrected.

