# Supplementary material for: Epilepsy and neuropsychiatric comorbidities in mice carrying a recurrent Dravet syndrome SCN1A missense mutation
Source: Sci Rep. 2019 Oct 2;9:14172. doi: 10.1038/s41598-019-50627-w (PMC6775062; doi:10.1038/s41598-019-50627-w)
Supplement: Supplementary file 1 — Full-lenght western blots [file 41598_2019_50627_MOESM1_ESM.pdf]

Epilepsy and neuropsychiatric comorbidities in mice carrying a recurrent Dravet syndrome *SCN1A* missense mutation

Ana Ricobaraza[1*], Lucia Mora-Jimenez[1], Elena Puerta[2], Rocio Sanchez-Carpintero[3], Ana Mingorance[4], Julio Artieda[5], Maria Jesus Nicolas[6], Guillermo Besne[6], Maria Bunuales[1], Manuela Gonzalez-Aparicio[1], Noemi Sola-Sevilla[3], Miguel Valencia[6&], Ruben Hernandez-Alcoceba[1&]

1. University of Navarra. Gene Therapy Program CIMA. IdiSNA, Navarra institute for health research. Pamplona, Spain.
2. University of Navarra. Department of Pharmacology and Toxicology. IdiSNA, Navarra institute for health research. Pamplona, Spain.
3. University Clinic of Navarra. Dravet Syndrome Unit. Pediatric Neurology Unit. IdiSNA, Navarra institute for health research. Pamplona, Spain.
4. Dracaena Consulting. Madrid, Spain.
5. University of Navarra. Neuroscience Program CIMA. IdiSNA, Navarra institute for health research. Neurophysiology Service, Clinica Universidad de Navarra, University of Navarra. Pamplona, Spain.
6. University of Navarra. Neuroscience Program CIMA. IdiSNA, Navarra institute for health research. Pamplona, Spain.

*Corresponding author: aricobaraza@unav.es

[&]These authors share senior authorship.

Keywords:

Dravet Syndrome, SCN1A, Nav1.1, epilepsy, gene therapy, adenovirus, encephalopathy.

Western blot

In each image the first set of blots corresponds to mouse Nav1.1 (**a**) and the second one to mouse GAPDH (**b**).

Analyzed regions:
1. Total cortex.
2. Hippocampus.
3. Cerebellum.
4. Prefrontal cortex. The analysis of this region was omitted to simplify the general message of the paper since no differences were observed compared to total cortex.

Samples were loaded into the gels as follows:
- Scn1a$_{WT/WT}$ (n=3).
- Scn1a$_{WT/A1783V}$ (n=5).
- Scn1a$_{WT/WT}$ (n=3).
- Scn1a$_{WT/A1783V}$ (n=3).

In the final figure, blots were cropped and rearranged to display together Scn1a$_{WT/WT}$ and Scn1a$_{WT/A1783V}$ mice.

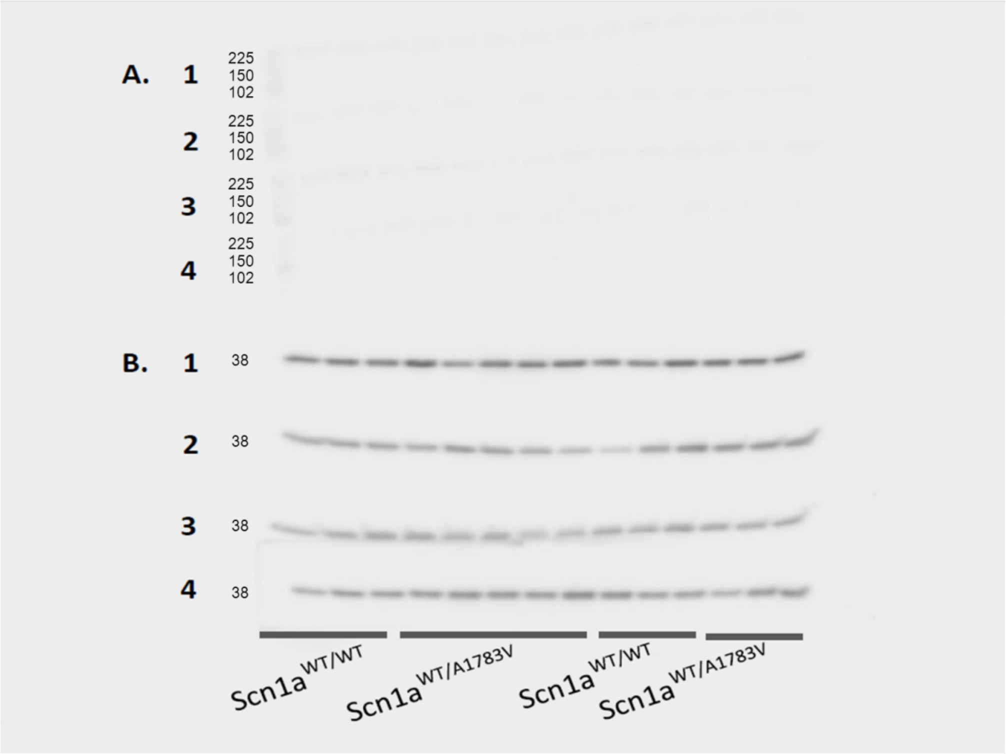

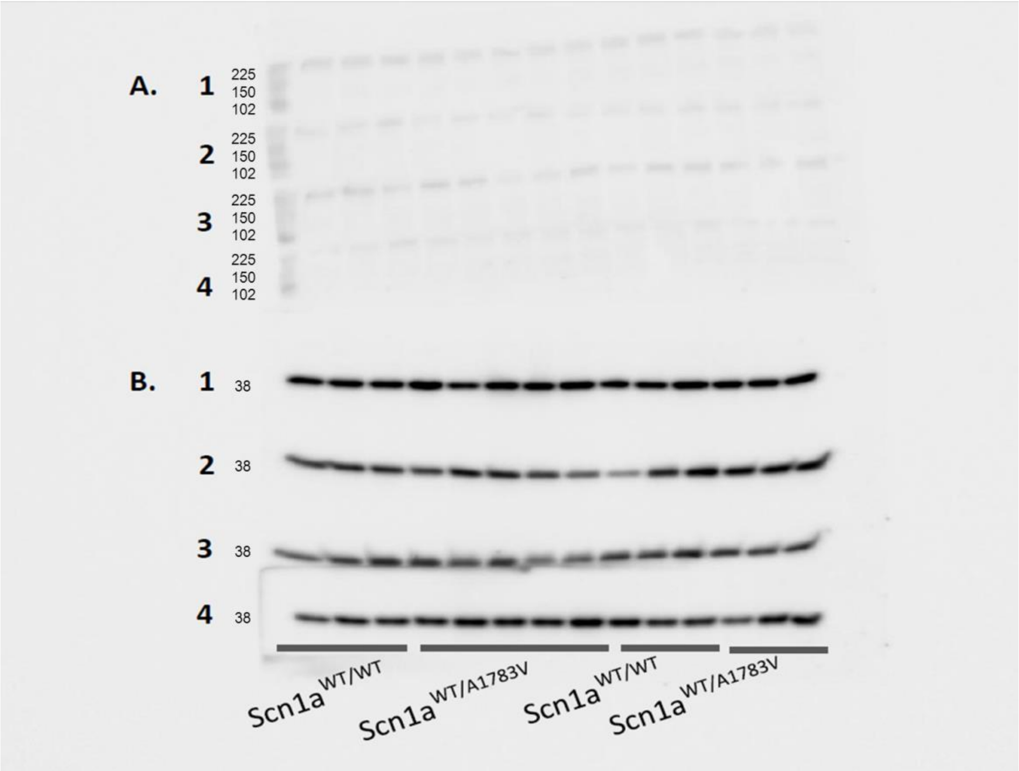

*Image employed for the densitometric analysis of GAPDH expression.

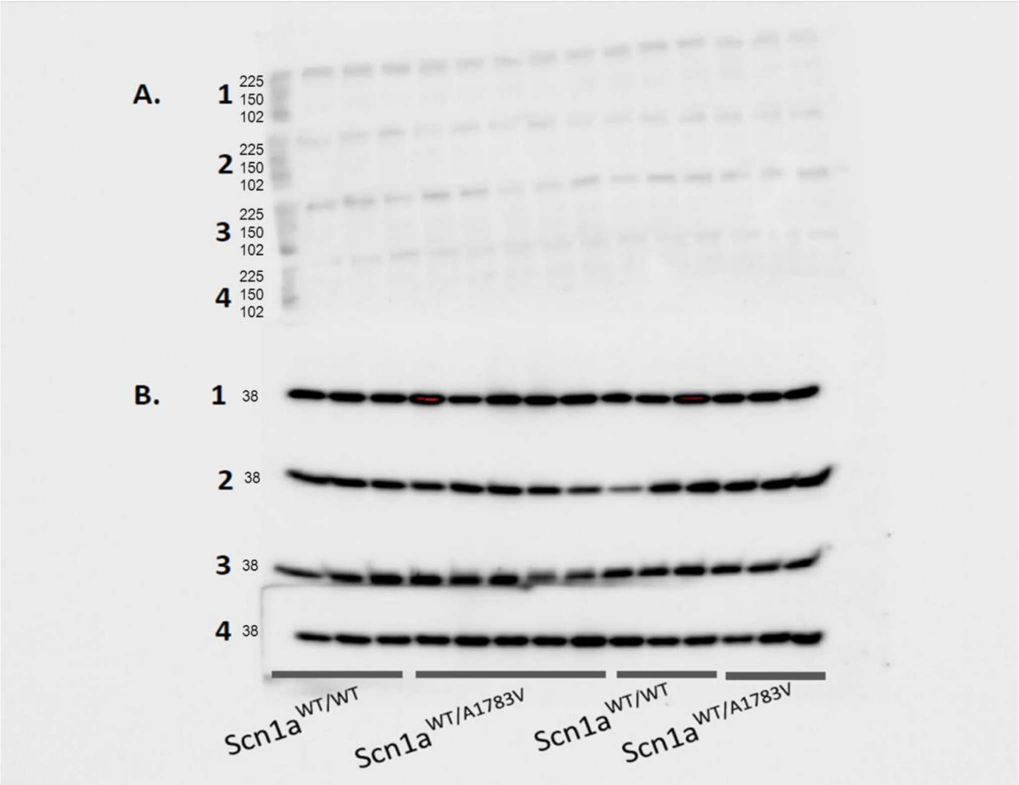

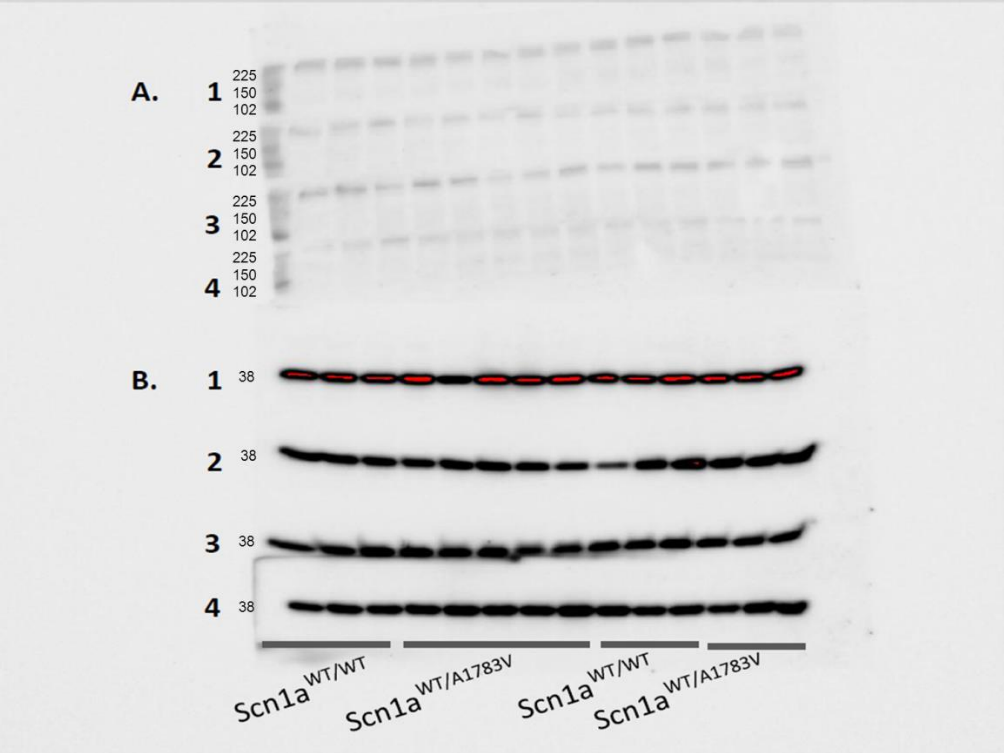
A.
1
225
150
102
2
225
150
102
3
225
150
102
4
225
150
102
B.
1
38
2
38
3
38
4
38
Scn1a WT/WT
Scn1a WT/A1783V
Scn1a WT/WT
Scn1a WT/A1783V

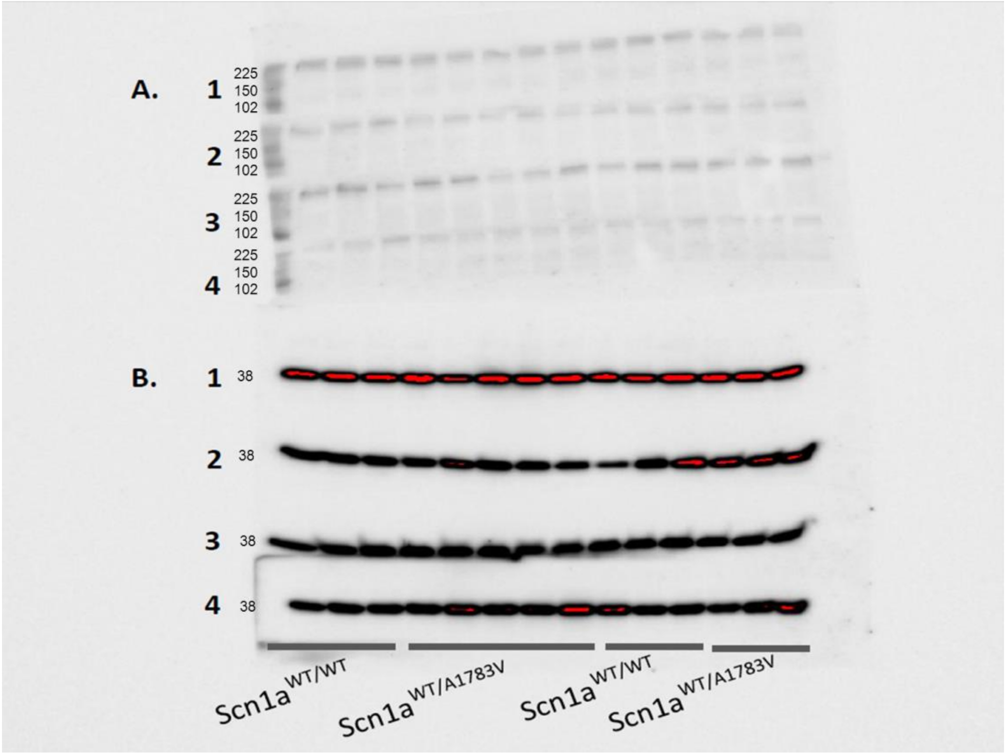
A.
1
225
150
102
2
225
150
102
3
225
150
102
4
225
150
102
B.
1
38
2
38
3
38
4
38
Scn1a WT/WT
Scn1a WT/A1783V
Scn1a WT/WT
Scn1a WT/A1783V

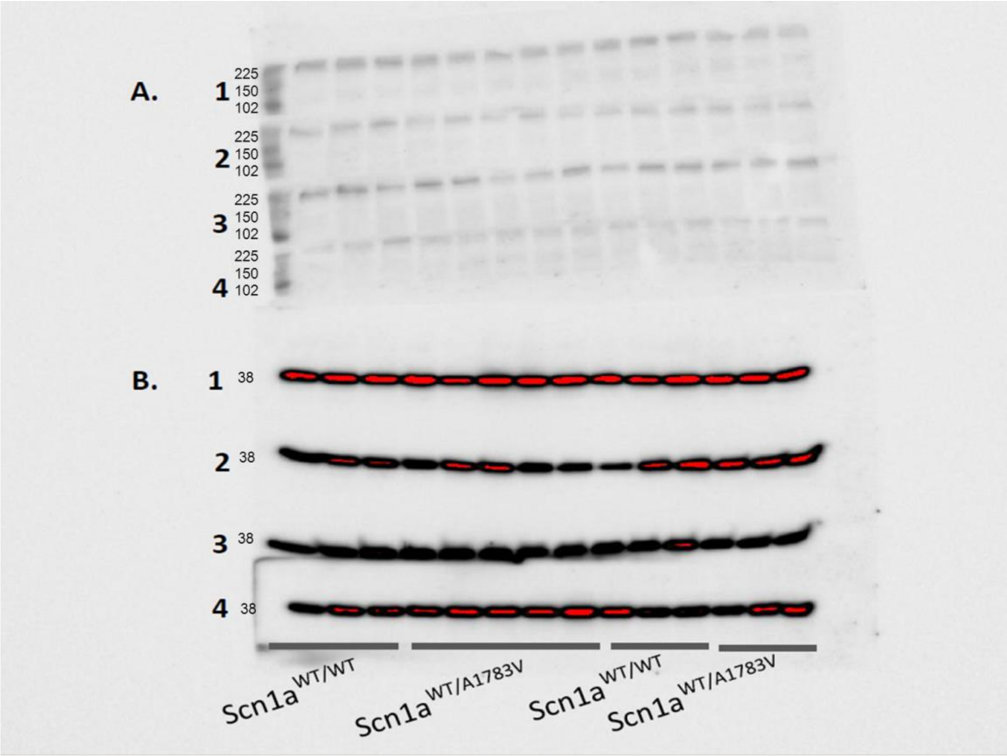

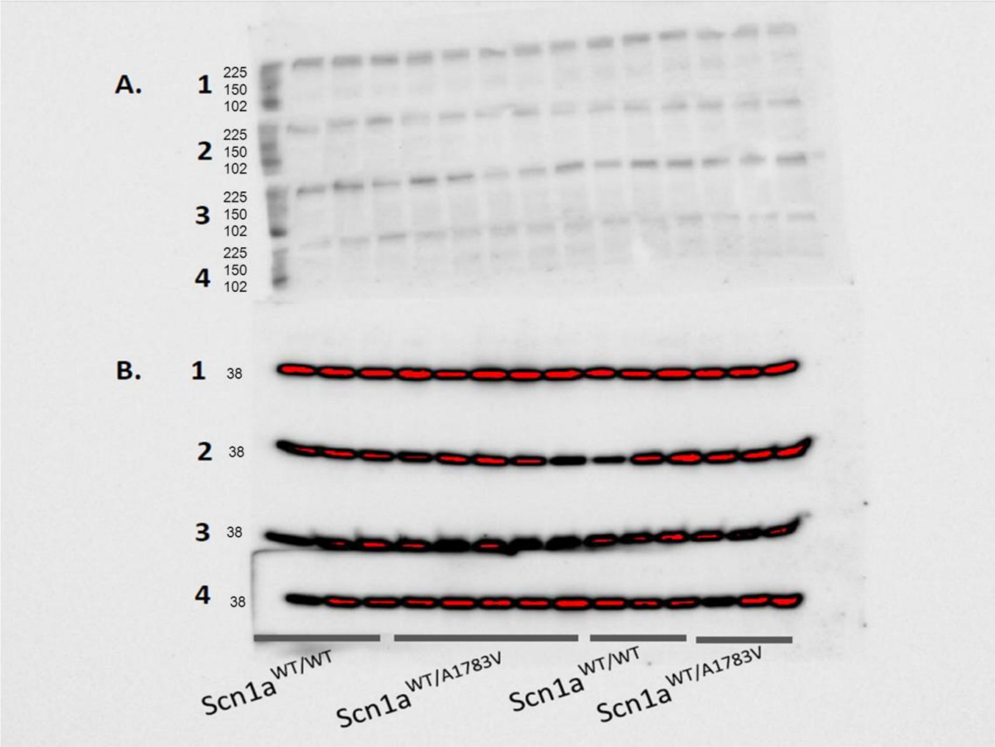

*Image employed for the densitometric analysis of Nav1.1 expression.
